# A novel *Drosophila* model of TDP-43 proteinopathies: N-terminal sequences combined with the Q/N domain induce protein functional loss and locomotion defects

**DOI:** 10.1242/dmm.023382

**Published:** 2016-06-01

**Authors:** Simona Langellotti, Valentina Romano, Giulia Romano, Raffaella Klima, Fabian Feiguin, Lucia Cragnaz, Maurizio Romano, Francisco E. Baralle

**Affiliations:** 1 International Centre for Genetic Engineering and Biotechnology, Padriciano 99, Trieste I-34149, Italy; 2 Department of Life Sciences, University of Trieste, Via A. Valerio 28, Trieste 34127, Italy

**Keywords:** TDP-43, TBPH, *Drosophila*, ALS, FTLD, N-terminus, Aggregation

## Abstract

Transactive response DNA-binding protein 43 kDa (TDP-43, also known as TBPH in *Drosophila melanogaster* and TARDBP in mammals) is the main protein component of the pathological inclusions observed in neurons of patients affected by different neurodegenerative disorders, including amyotrophic lateral sclerosis (ALS) and fronto-temporal lobar degeneration (FTLD). The number of studies investigating the molecular mechanisms underlying neurodegeneration is constantly growing; however, the role played by TDP-43 in disease onset and progression is still unclear. A fundamental shortcoming that hampers progress is the lack of animal models showing aggregation of TDP-43 without overexpression. In this manuscript, we have extended our cellular model of aggregation to a transgenic *Drosophila* line. Our fly model is not based on the overexpression of a wild-type TDP-43 transgene. By contrast, we engineered a construct that includes only the specific TDP-43 amino acid sequences necessary to trigger aggregate formation and capable of trapping endogenous *Drosophila* TDP-43 into a non-functional insoluble form. Importantly, the resulting recombinant product lacks functional RNA recognition motifs (RRMs) and, thus, does not have specific TDP-43-physiological functions (i.e. splicing regulation ability) that might affect the animal phenotype per se. This novel *Drosophila* model exhibits an evident degenerative phenotype with reduced lifespan and early locomotion defects. Additionally, we show that important proteins involved in neuromuscular junction function, such as syntaxin (SYX), decrease their levels as a consequence of TDP-43 loss of function implying that the degenerative phenotype is a consequence of TDP-43 sequestration into the aggregates. Our data lend further support to the role of TDP-43 loss-of-function in the pathogenesis of neurodegenerative disorders. The novel transgenic *Drosophila* model presented in this study will help to gain further insight into the molecular mechanisms underlying neurodegeneration and will provide a valuable system to test potential therapeutic agents to counteract disease.

## Abstract (precis)

**Summary:** An engineered TDP-43 construct can be used to induce TDP-43 aggregation in *Drosophila*, providing a model that could be useful for characterization of pathogenetic mechanisms and drug screening.

## INTRODUCTION

Fronto-temporal lobar degeneration (FTLD) and amyotrophic lateral sclerosis (ALS) are two neurodegenerative diseases that can exist both as distinct clinical entities and as a clinical continuum with overlapping pathogenic pathways (Van Langenhove et al., 2012). A common feature of these diseases are cytoplasmic aggregates of TDP-43 (also known as TBPH in *Drosophila melanogaster* and TARDBP in mammals), resulting in nuclear clearance of the protein (Belzil et al., 2013). TDP-43 is a heterogeneous nuclear ribonucleoprotein (hnRNP) with nuclear and cytoplasmic functions (Buratti and Baralle, 2012) that are evolutionarily conserved from invertebrates to rodents and humans (Ayala et al., 2005). By exploiting the functional overlap between the human (h-) and fruit fly (d-) TDP-43 orthologs (Ayala et al., 2005), different *Drosophila* models carrying the targeted disruption of the *TDP-43* gene have being generated and virtually all exhibit ALS-like neuromuscular deficits (Romano et al., 2012), indicating a loss of function of TDP-43 being central to the pathogenesis (Buratti and Baralle, 2009; Chen-Plotkin et al., 2010; Da Cruz and Cleveland, 2011; Lee et al., 2012). In accordance with this hypothesis, mutations identified within the *TARDBP* gene in ALS familial cases are in the C-terminal region and some seem to associate with enhanced TDP-43 aggregation (Arai et al., 2006; Neumann et al., 2006; Sreedharan et al., 2008). The C-terminal region of TDP-43 contains a Q- and N-rich region (denoted the Q/N region) involved in protein-protein interaction (D'Ambrogio et al., 2009), and it has been suggested that this sequence resembles a prion-like domain (Gitler and Shorter, 2011; Polymenidou and Cleveland, 2011). The Q/N region is crucial for the aggregation process, as demonstrated by different *in vitro* models (Igaz et al., 2009; Fuentealba et al., 2010; Budini et al., 2012b, 2015). In particular, we have shown that expression of 12 repetitions of the Q/N-rich amino acid sequence 331-369 of hTDP-43 (12×Q/N) fused to an EGFP tag (EGFP-12×Q/N) triggers the formation of aggregates that recapitulate the most relevant properties of the inclusions found in patients (Budini et al., 2012a,b).

Cells expressing EGFP-12×Q/N show colocalization of endogenous TDP-43 with the cytoplasmic aggregates induced by the transgene. However, no significant loss of TDP-43 function is observed. In a transgenic *Drosophila melanogaster* line expressing the construct EGFP-12×Q/N in the eye under the control of the GMR-Gal4 driver, it has been shown that EGFP-12×Q/N is able to trigger aggregation similarly to that observed in cells and that there is no intrinsic toxicity of the aggregates (Cragnaz et al., 2014). In a follow up of that study, the EGFP-12×Q/N construct was expressed as a transgene using the pan-neuronal elav-Gal4 driver. The transgenic fly presents a locomotion defect phenotype in mid-adult life, coinciding with a physiological and age-related fourfold reduction of dTDP-43 levels (Cragnaz et al., 2015), whereas no significant changes in the expression of dTDP-43-regulated genes are detected in these animals (L. Cragnaz, personal communication). This observation suggests that, although trapping of endogenous TDP-43 into EGFP-12×Q/N aggregates occurs, it is not highly efficient using this transgene. Consequently the phenotypic onset is detected only when endogenous dTDP-43 levels drop.

Further studies in tissue culture cells have shown that in addition to the TDP-43 C-terminal region, the 1-75 N-terminal portion of TDP-43 is crucial to triggering the formation of aggregates able to efficiently trap endogenous TDP-43 in a non-functional insoluble form (Budini et al., 2015; Romano et al., 2015). These results prompted us to produce a novel transgenic *Drosophila* line based on a construct carrying the N-terminal domain of TDP-43 in addition to the 12×Q/N repetitions. The transgene induced an efficient loss of function of endogenous TDP-43 in *Drosophila* in a similar way to that observed in human tissue culture cells. The transgenic fly also exhibited an evident degenerative phenotype, with reduced lifespan and early locomotion defects.

## RESULTS

### Generation of a novel construct to model TDP-43 aggregation

**Fig. 1.**
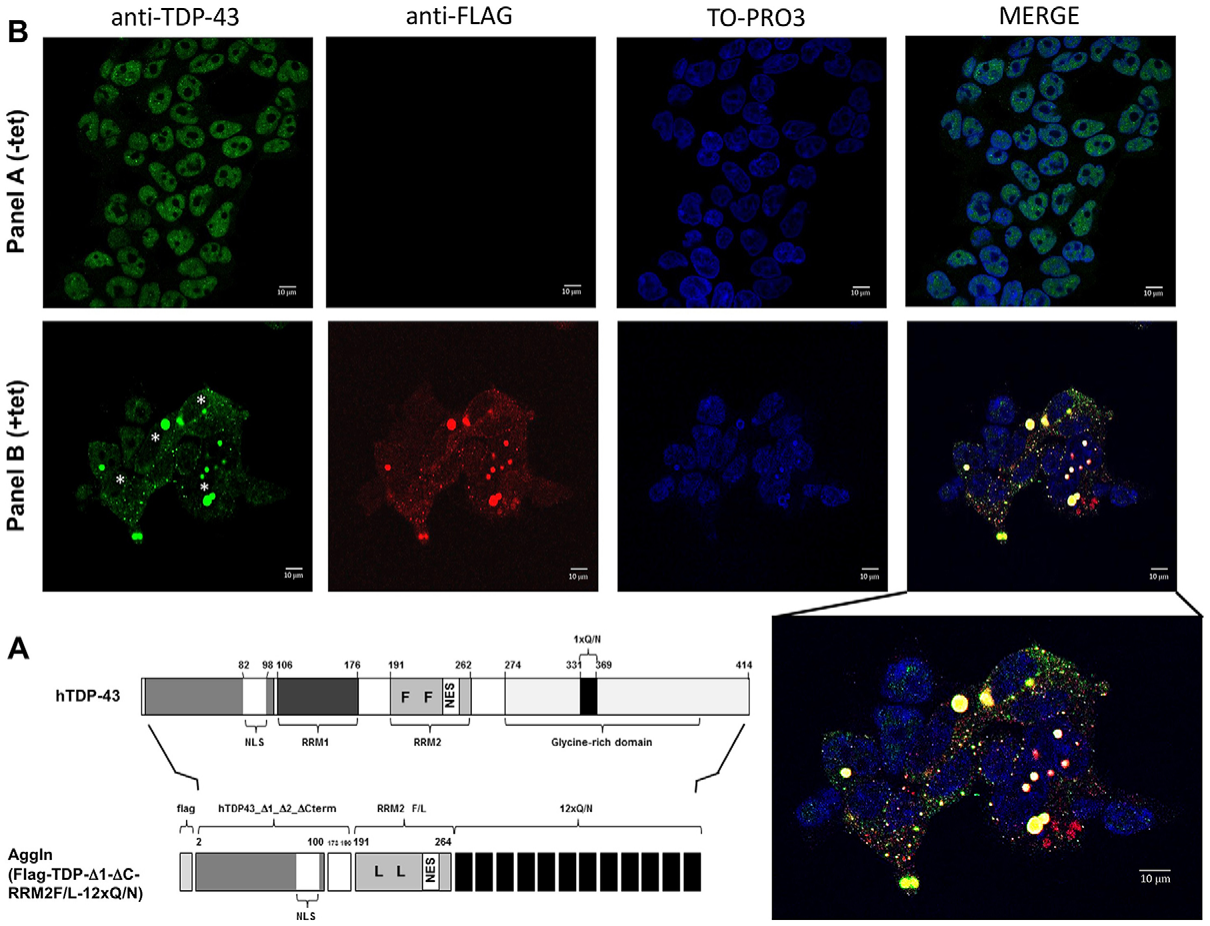
**Schematic representation of the AggIn construct and immunofluorescence of the HEK293 AggIn stable cell line.** (A) All relevant elements within the AggIn (Flag-TDP-Δ1-ΔC-RRM2F/L-12×Q/N) construct are identified along with their relative position in the wild-type human TDP-43 protein (hTDP-43). (B) Panel A shows HEK293 AggIn cells without tetracycline (-tet) induction and Panel B shows HEK293 AggIn cells after tetracycline induction (+tet). Anti-Flag immunofluorescence is visualized as red fluorescence, whereas anti-TDP-43 immunofluorescence is visualized as green fluorescence. Cell nuclei were stained with the reagent TO-PRO3 (blue). Empty nuclei in AggIn-expressing cells are marked with asterisks. A merge between Flag, TDP-43 and TO-PRO3 staining is shown. A higher magnification of the +tet merge panel is also reported. Scale bars: 10 μm.

We have previously shown that expression of the 1-75 N-terminal portion of TDP-43 fused to 12 tandem repeats of its prion-like Q/N-rich region (12×Q/N) in a cell line is able to trigger the aggregation of endogenous TDP-43, resulting in its loss of function as determined by POLDIP3 exon 3 alternative splicing (Budini et al., 2015). In this manuscript, we designed a novel construct to make a chimeric protein (Fig. 1A) that harbors the entire 1-100 N-terminal domain of the TDP-43 protein (including the nuclear localization sequence, NLS), the linker region between the two RNA recognition motifs (RRMs), a mutated form of the RRM2 unable to bind RNA but that retains the nuclear export sequence (NES; RRM2F/L), and, finally, the 12×Q/N repeats. The features of this transgene, Flag-TDP-Δ1-ΔC-RRM2F/L-12×Q/N [denoted aggregation inducer (AggIn)], provide a reasonable degree of structural integrity to the chimeric protein and include both the NLS and the NES that ensure the preservation of TDP-43 shuttling abilities between nucleus and cytoplasm. For technical convenience, we also included an N-terminal Flag tag.

### Characterization of a HEK293 AggIn stable cell line

**Fig. 2.**
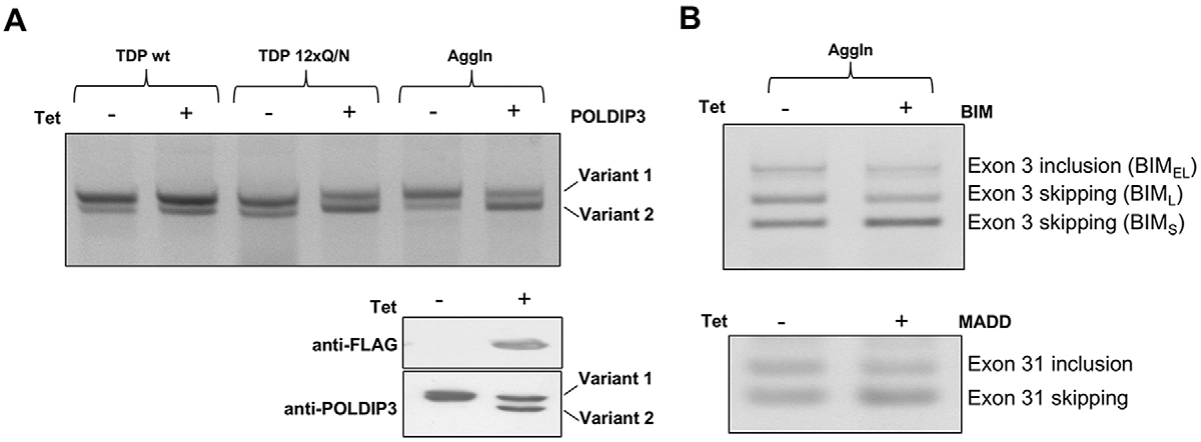
**Effect of transgene expression on TDP-43 target genes in the HEK293 AggIn stable cell line.** (A) Upper panel, RT-PCR showing the splicing pattern of the endogenous POLDIP3 gene (exon 3) after tetracycline (tet) induction (+) or not (−) of Flag-tagged wild-type TDP-43 (TDP wt; left-hand two lanes), Flag-tagged TDP-12×Q/N (middle two lanes) or AggIn (right-hand two lanes) in stable HEK293 cell lines. Lower panel, western blot analysis, using anti-POLDIP3 antibody, of total protein lysates extracted from the same samples used in the two right-hand lanes of the upper panel. (B) RT-PCR showing the splicing pattern of endogenous BIM (exon 3) and MADD (exon 31) genes after tetracycline induction (+) or not (−) of the AggIn stable HEK293 cell line.

To test the aggregation efficiency of the transgene, we produced a HEK293 AggIn stable cell line. After tetracycline induction, anti-Flag staining showed the presence of many, prevalently cytosolic, aggregates (Fig. 1B, panel B, anti-FLAG +tet). Interestingly, several cell nuclei appeared to be devoid of endogenous TDP-43 (Fig. 1B, Panel B, anti-TDP-43 +tet; empty nuclei marked with asterisks). In order to analyze whether the formation of these aggregates was matched by loss of TDP-43 function, we evaluated the splicing profile of the endogenous gene POLDIP3, whose pre-mRNA processing is determined by TDP-43. In fact, knockdown of TDP-43 causes the exclusion of exon 3 from the mature POLDIP3 mRNA (variant 2) (Fiesel et al., 2012; Shiga et al., 2012). Similar to what happens with overexpression of the TDP-12×Q/N construct (Fig. 2A, upper panel, middle two lanes) (Budini et al., 2015), tetracycline induction of the AggIn protein expression was associated with a strong increase of POLDIP3 variant 2 at both the mRNA (Fig. 2A, upper panel, right-hand two lanes) and protein levels (Fig. 2A, lower panel). This effect is specific for constructs able to induce aggregation and efficient trapping of endogenous TDP-43: in fact, no alteration in the POLDIP3 splicing pattern was observed following overexpression of wild-type TDP-43 (Fig. 2A, upper panel, left-hand two lanes) or, as previously reported, of EGFP-12×Q/N (Budini et al., 2012b, 2015).

Furthermore, we also performed the splicing assay on the two additional endogenous transcripts BIM and MADD, whose splicing profile is known to be affected upon TDP-43 depletion (De Conti et al., 2015). For both genes, the splicing profiles of the transcripts in the HEK AggIn stable cell line resembled changes previously observed upon TDP-43 silencing (Fig. 2B; increased BIM exon 3 and MADD exon 31 skipping).

Taken together, these experiments provide evidence of the endogenous TDP-43 loss of function upon AggIn expression and indicate that AggIn is a promising construct to model TDP-43 aggregation *in vivo* in *Drosophila melanogaster*.

### Pan-neuronal expression of the AggIn construct in *Drosophila melanogaster*

**Fig. 3.**
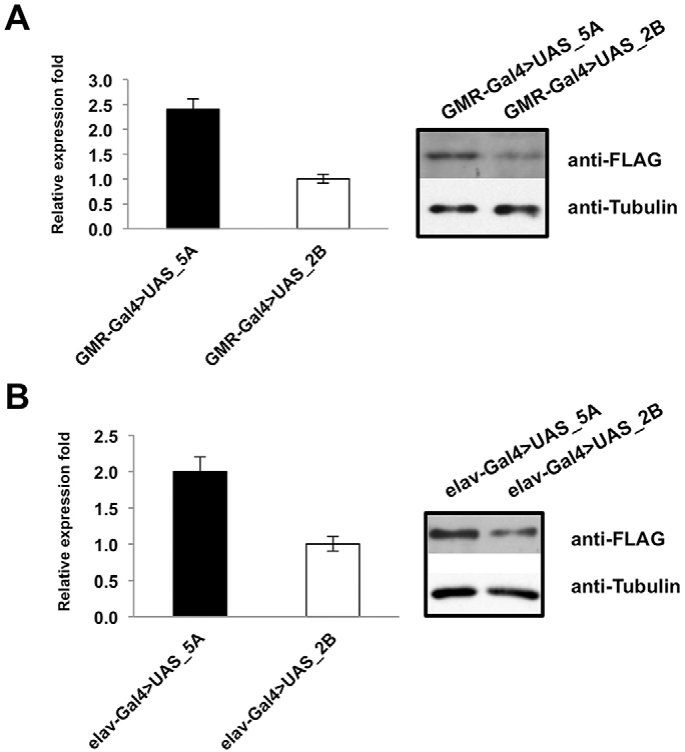
**Expression levels of transgene in two *Drosophila* lines.** (A) Western blot analysis of total protein extracts from fly heads of GMR-Gal4>UAS_5A and GMR-Gal4>UAS_2B and (B) elav-Gal4>UAS_5A and elav-Gal4>UAS_2B lines. Eye-specific and pan-neuronal expression of the AggIn construct was achieved using the GMR- and elav-Gal4 drivers, respectively. Western blot densitometry was performed using the ImageJ software and the normalized expression of the transgenic protein is reported in the graphs (mean±s.e.m., *n*=3).

To create a novel animal model for TDP-43 aggregation, we cloned the AggIn construct in the pUASTattB vector, under the control of the upstream activating sequence (UAS). After embryo injection, five different fly lines were obtained and screened for transgene expression by using the GMR-Gal4 driver. Although four of these lines expressed the transgene at comparable levels, the fifth demonstrated a higher expression level (data not shown). Therefore, in the subsequent steps of our study, we focused our attention on two of these transgenic lines: the one expressing the transgene at the top expression level (UAS_5A) and one out of the four expressing the transgene at comparable level (UAS_2B) (Fig. 3A).

To start studying the effects of transgene expression on the *Drosophila* phenotype, we expressed the construct selectively in neurons, by crossing the UAS_5A and UAS_2B flies with a fly containing the pan-neuronal elav-Gal4 driver (the flies were grown at 25°C). As expected, elav-Gal4>UAS_5A flies demonstrated a transgene expression level twofold higher than the one observed in elav-Gal4>UAS_2B, as calculated from normalized expression values (Fig. 3B).

### Fly survival is dramatically affected upon AggIn expression in neurons

**Fig. 4.**
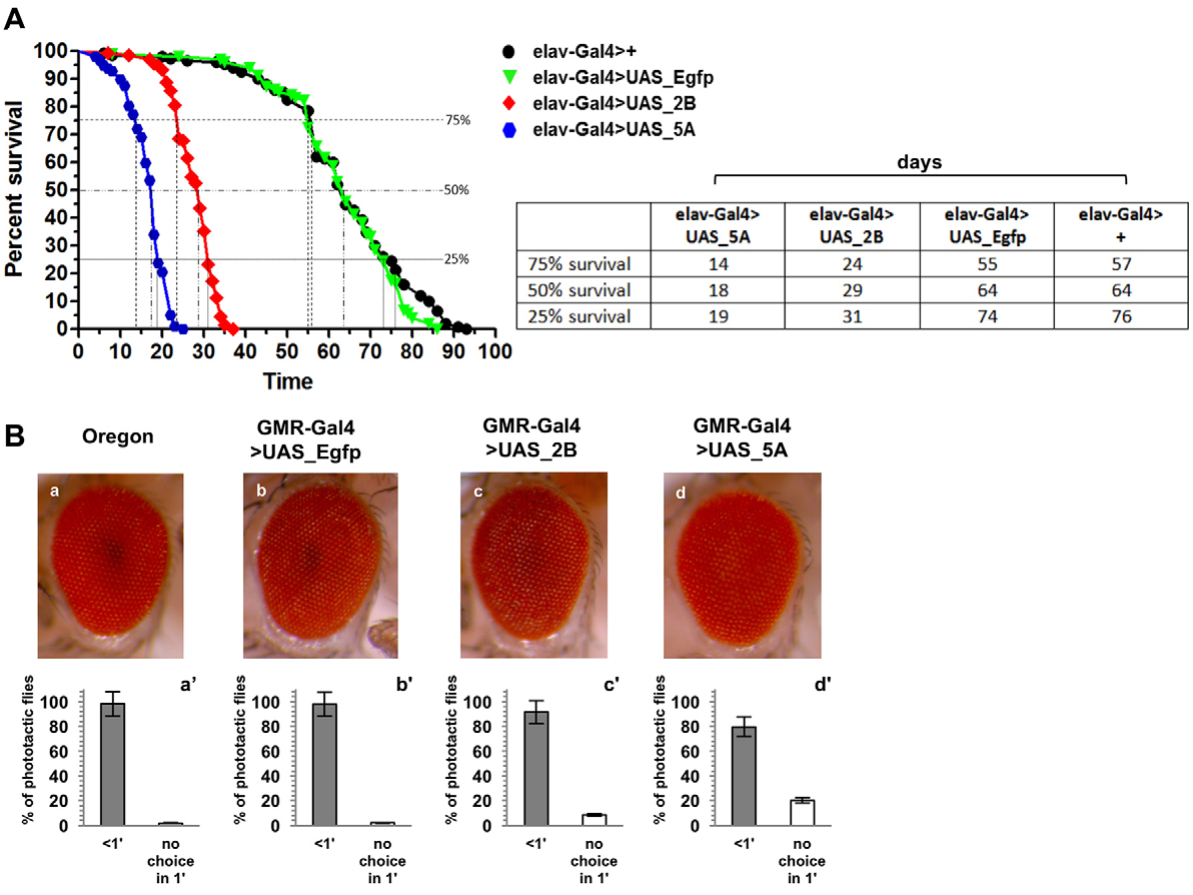
**Effect of transgene expression on *Drosophila* lifespan and on external eye structure and/or function.** (A) Lifespan is dramatically reduced in flies expressing the AggIn transgene in neurons (elav-Gal4>UAS_5A and elav-Gal4>UAS_2B) versus a control fly not expressing any transgene (elav-Gal4>+) or a transgenic fly line expressing the control protein EGFP (elav-Gal4>UAS_Egfp). Median survival is 18 days for elav-Gal4>UAS_5A; 29 days for elav-Gal4<UAS_2B; 64 days for controls (both elav-Gal4>+ and elav-Gal4>UAS_Egfp). *n*>120 animals for each genotype; *P*<0.001 (log-rank test) for all the following genotype pairs: elav-Gal4>+ versus elav-Gal4>UAS_2B; elav-Gal4>+ versus elav-Gal4>UAS_5A; elav-Gal4>UAS_Egfp versus elav-Gal4>UAS_2B; elav-Gal4>UAS_Egfp versus elav-Gal4>UAS_5A; elav-Gal4>UAS_2B versus elav-Gal4>UAS_5A. Time points (days) corresponding to 25%, 50% and 75% survival are also shown in the graph for each genotype and reported in the flanking summary table. (B) External eye phenotype and phototaxis assay of 1-day-old flies. (a,a′) Oregon (wild-type); (b,b′) GMR-Gal4>UAS_Egfp; (c,c′) GMR-Gal4>UAS_2B; (d,d′) GMR-Gal4>UAS_5A. The AggIn expression in the eye, using the GMR-Gal4 driver, did not result in any substantial alteration of the external eye phenotype (upper panel, compare pictures in c and d versus controls in a and b). However, the vision assay (lower panel) revealed that a minor fraction of the AggIn-expressing population of flies (8.5% of GMR-Gal4>UAS_2B, and 20.4% of GMR-Gal4>UAS_5A) exhibited vision defects because they did not reach the light within 1 min of time in a phototaxis assay (compare white bars in c′ and d′ fractions with the negative controls in a′ and b′). Error bars indicate s.e.m. (*n*=3).

To study the effects of the AggIn expression in neurons, we first analyzed the lifespan of elav-Gal4>UAS_5A and elav-Gal4>UAS_2B flies versus two different control flies: one not expressing any transgene (elav-Gal4>+), and a second transgenic fly line expressing the irrelevant protein EGFP (elav-Gal4>UAS_Egfp). As clearly shown in Fig. 4A, lifespan was dramatically reduced in flies expressing AggIn. Indeed, whereas we observed a median survival of 64 days for control flies (both elav-Gal4>+ and elav-Gal4>UAS_Egfp), we found a median survival of only 18 days for elav-Gal4>UAS_5A and 29 days for elav-Gal4<UAS_2B. Such a significant decline in survival during aging suggests that transgene expression has strong phenotypic consequences, whose intensity is related to transgene expression levels.

The intrinsic toxicity of the protein aggregates was low. In fact, whereas the expression of a wild-type dTDP-43 transgene using the GMR-Gal4 driver resulted in the formation of large necrotic patches in the eye of newly eclosed flies (Fig. S1A), the expression of the AggIn transgene did not substantially alter the eye anatomy, although there was a modest change in visual ability, particularly in the high-expression UAS_5A line (Fig. 4B). Furthermore, we also analyzed the external eye phenotype of 15-day-old transgenic flies, and did not observe any appreciable anatomic difference nor increased signs of toxicity in comparison to the 1-day-old eye (Fig. 4B; Fig. S1B). This suggests that AggIn expression also does not induce obvious signs of eye-structure degeneration during aging. Therefore, both fly lines were considered suitable for further studies of the phenotypic effects of aggregation.

### Transgene expression affects climbing ability of flies

We then investigated whether a locomotion defect appears at some point of the reduced lifespan of these flies using the climbing ability test.

**Fig. 5.**
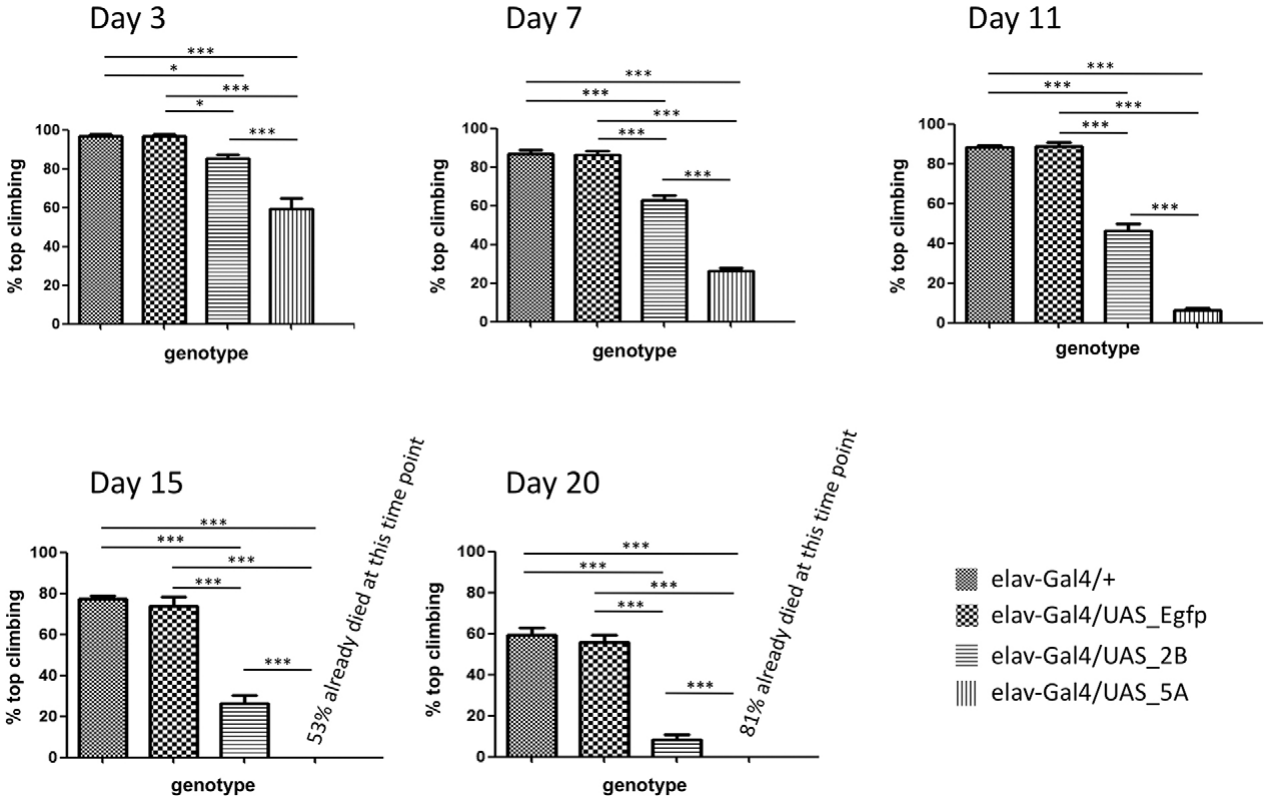
**Effect of transgene expression on *Drosophila* climbing ability.** A climbing assay performed in flies expressing the AggIn transgene (elav-Gal4>UAS_5A and elav-Gal4>UAS_2B) versus a control fly not expressing any transgene (elav-Gal4>+) and a transgenic fly line expressing the control protein EGFP (elav-Gal4>UAS_Egfp). A statistically significant impairment of climbing is already observed at day 3 in both the AggIn-expressing fly lines. The climbing defect gets worse with age, from day 3 to 20. *P<0.05, ***P<0.001 (one-way ANOVA). Error bars indicate s.e.m. (*n*>120 animals for each genotype).

We assayed the flies at five different time-points after eclosion (days 3, 7, 11, 15 and 20). Both elav-Gal4>UAS_5A and elav-Gal4>UAS_2B flies already demonstrated a statistically significant impairment of the climbing ability at the first time-point (day 3). As expected, at each time point analyzed, the elav-Gal4>UAS_5A showed a more severe impairment than the elav-Gal4>UAS_2B (Fig. 5). Indeed, whereas ∼40% of elav-Gal4>UAS_5A flies were no longer able to reach the top of the cylinder at day 3, only 15% of elav-Gal4>UAS_2B flies demonstrated a similar impairment of climbing at this time point. Similarly, more than 70% of elav-Gal4>UAS_5A flies were no longer able to reach the top of the tube 7 days after eclosion, whereas a similar percentage of elav-Gal4>UAS_2B flies with impaired climbing ability was observed only after another 8 days (day 15 after eclosion).

Taken together, these results suggest that the AggIn product affects the motility of flies in an expression-dependent manner.

### Pan-neuronal expression of the transgene in elav-Gal4>UAS_5A results in early locomotion impairment, which is detectable in the larval stage

**Fig. 6.**
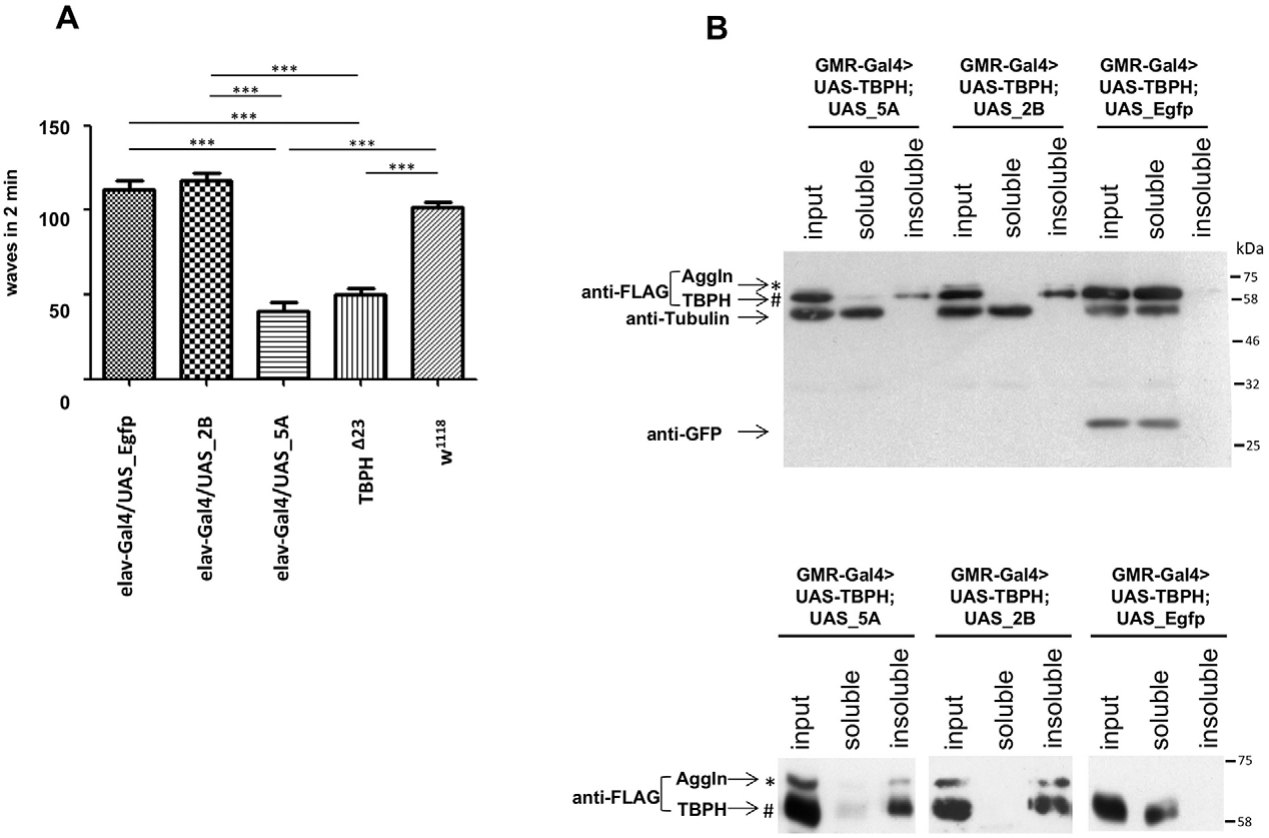
**Effect of transgene expression on *Drosophila* larval motility and solubility assay on adult fly heads.** (A) A larval motility assay was performed on third-instar larvae. A strong reduction in larval motility of elav-Gal4>UAS_5A (5A) larvae is observed, as compared to a transgenic line expressing the control protein EGFP (elav-Gal4>UAS_Egfp) and to the wild-type line (w^1118^). No impairment in larval motility is observed in elav-Gal4>UAS_2B larvae (2B). A TDP-43-null allele line (TBPH^Δ23^) was used as a positive reference control. *x*-axis, genotype; *y*-axis, peristaltic waves counted in two minutes. Error bars indicate s.e.m. (*n*=20 animals for each genotype). ***P<0.001 (one-way ANOVA). (B) Solubility assay. Western blot of fractionated proteins obtained from adult fly heads of the following genotypes: GMR-Gal4>UAS_TBPH; UAS_5A, GMR-Gal4>UAS_TBPH; UAS_2B, GMR-Gal4>UAS_TBPH; UAS_Egfp (TBPH is the *Drosophila* TDP-43). Upper panel, input, soluble and insoluble fractions of each genotype were loaded in a 1:1:1 ratio and probed by immunoblotting. AggIn and TBPH were detected with anti-FLAG antibody. EGFP was detected using anti-GFP antibody. Anti-tubulin served as protein loading control. Lower panel, to improve the separation of Flag-AggIn (see *) and Flag-TBPH (see #) protein bands, which have a close molecular mass, the three sample fractions from each genotype were also loaded on additional gels and were run for a longer time, before anti-Flag immunoblotting. TBPH is mostly insoluble when it is co-expressed with AggIn; by contrast, it remains mainly soluble when it is co-expressed with the unrelated protein EGFP.

The possibility of an early lethality of the AggIn flies was checked: we selected third-instar larvae and transferred them to fresh food tubes. After 6 days, we calculated the percentages of eclosed flies, pupal lethality and larval lethality (Fig. S2). Whereas the elav-Gal4>UAS_2B line did not show differences in larval lethality compared to controls (elav-Gal4>+, elav-Gal4>UAS_Egfp) and revealed only a slight increase in pupal lethality, elav-Gal4>UAS_5A animals demonstrated a higher larval and pupal lethality compared to both the controls and to the elav-Gal4>UAS_2B line. Nonetheless, we were able to analyze the phenotype of the animals during the larval stage. To this aim, we assayed third-instar larvae movement by counting the number of their peristaltic waves in 2 min on a suitable solid substrate (see Materials and Methods for details). In addition, as a negative control of the experiment, we used a transgenic line expressing the irrelevant protein EGFP in neurons (elav-Gal4>UAS_Egfp) and the wild-type control line w^1118^. We also, as a positive control, analyzed the movement of TBPH^Δ23^ larvae, the first-discovered dTDP-43-null allele fly line, which shows a severe neurodegenerative phenotype with a locomotion defect in larval stages and dramatic locomotive defects after eclosion (Feiguin et al., 2009). We did not observe any significant difference in larval motility of the elav-Gal4>UAS_2B larvae with respect to the negative controls (Fig. 6A). The elav-Gal4>UAS_5A larvae, however, showed a significant motility impairment, as compared to the negative controls, with a reduced number of peristaltic waves, quantitatively comparable to those counted with dTDP-43-null larvae (TBPH^Δ23^) (Fig. 6A). Therefore, these results show that the locomotion impairment of the elav-Gal4>UAS_5A fly line is comparable with that of the dTDP-43-null model.

### Biochemical and functional assays support the notion of loss-of-function in endogenous *Drosophila* TDP-43 in transgenic AggIn fly models

The creation of the HEK293 AggIn stable cell line has shown that transgene expression efficiently triggers the formation of aggregates able to recruit and trap the endogenous TDP-43 protein and give rise to a TDP-43 loss-of-function effect (as demonstrated by the alteration of the splicing profile of the endogenous genes POLDIP3, BIM and MADD).

To show that AggIn expression also induces the formation of insoluble aggregates able to trap endogenous dTDP-43 in flies, we performed solubility experiments on transgenic fly heads co-expressing AggIn and a Flag-tagged form of dTDP-43 under the control of the GMR-Gal4 driver. As a control experiment, Flag-dTDP-43 was co-expressed with the unrelated protein EGFP. This experiment clearly shows that AggIn expression results in the formation of insoluble aggregates (Flag-AggIn, *; Fig. 6B) and induces a very strong shift of dTDP-43 from the soluble to the insoluble fraction (Flag-TBPH, #; Fig. 6B). As expected, the expression of control EGFP did not result in the formation of insoluble aggregates nor did it alter the solubility pattern of dTDP-43, which remained mainly soluble.

### Western blot analysis of SYX and CSP proteins expression

In order to investigate whether biochemical evidence of dTDP-43 loss of function could also be found in the transgenic flies, we analyzed the expression levels of genes known to be altered in dTDP-43-null fly models and previously characterized as molecular targets of dTDP-43 potentially related to neurodegeneration pathogenesis.

In particular, we focused our attention on the elav-Gal4>UAS_5A line, which showed a striking degenerative phenotype during both adulthood and larval stage.

**Fig. 7.**
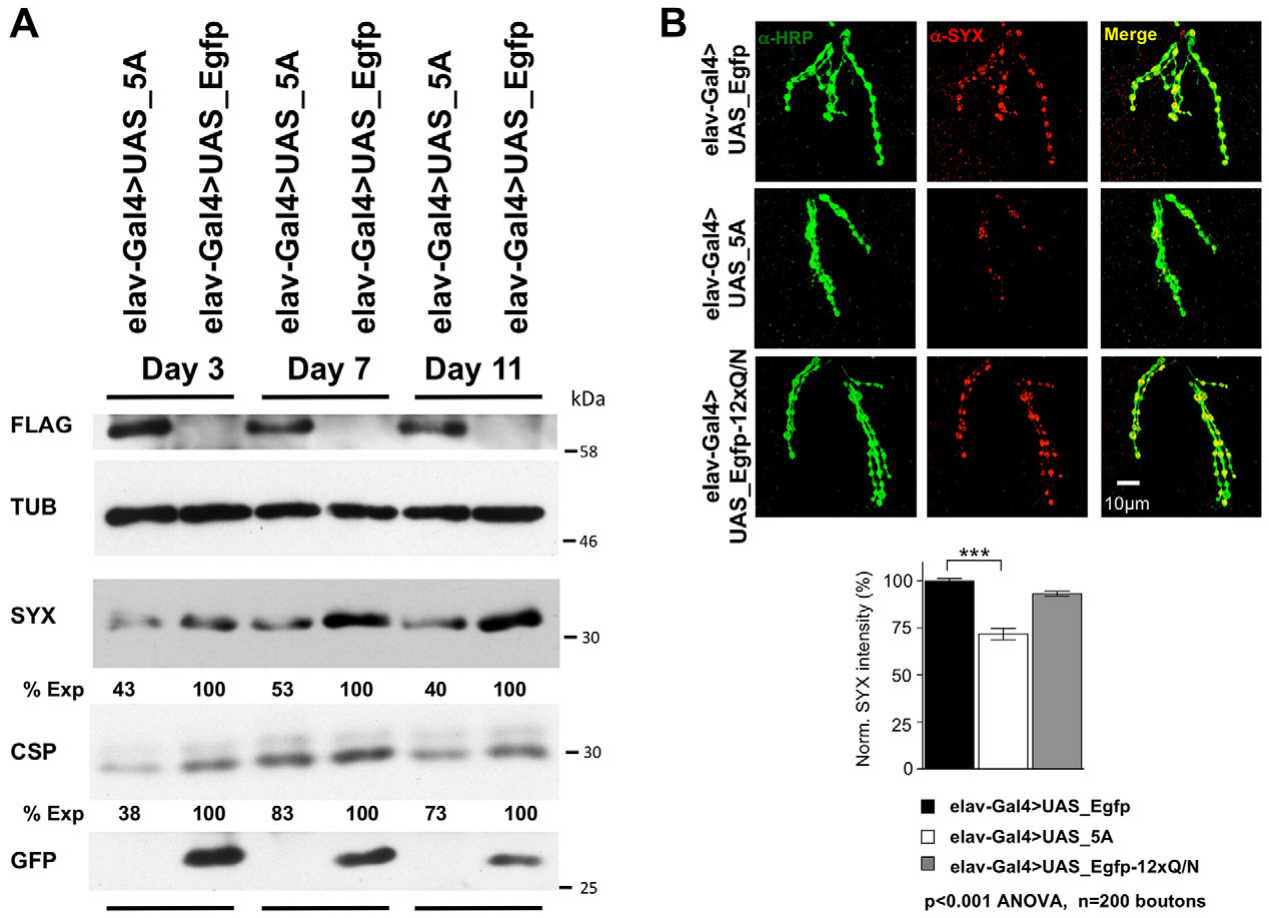
**Transgene effects on expression of TDP-43 target genes.** (A) Western blot for TDP-43-target genes performed in adult transgenic flies. Western blot analysis with anti-SYX and anti-CSP antibodies demonstrates the specific drop in expression of these presynaptic vesicular markers in elav-Gal4>UAS_5A fly heads, as compared to a transgenic line expressing the control protein EGFP (elav-Gal4>UAS_Egfp). Three different time-points were assayed (days 3, 7 and 11). Total protein samples were extracted from adult heads; anti-α-tubulin (TUB) was used as a loading control. The relative expression of target proteins in transgenic lines was calculated by optical densitometry with ImageJ software (Schneider et al., 2012). For each time-point, the percentage expression of SYX and CSP in the 5A line versus the control EGFP was calculated and is reported below the western blot panel. At day 3, the expression of SYX in 5A line was 43±15% that of the Egfp line; at day 7, it was 53±18; at day 11, it was 40±15%. At day 3, the expression of CSP in 5A line was 38±15% that of the Egfp line; at day 7, it was 83±8% s.e.m.; at day 11, it was 73±10% (results are mean±s.e.m.). All results shown are representative of at least three independent experiments. (B) Anti-syntaxin immunofluorescence performed in third-instar larvae NMJs. Confocal microscopy images of NMJs presynaptic boutons in muscle 6 and 7, II segment, are shown. SYX expression (anti-SYX antibody, red) appears to be strongly reduced in the synaptic terminals of elav-Gal4>UAS_5A larvae compared to elav-Gal4>UAS_Egfp control larvae. By contrast, the anti-HRP staining of the neurons is not affected (anti-HRP antibody, green). No significant alteration of the SYX protein expression was detected in elav-Gal4>UAS_Egfp-12×Q/N synaptic terminals. The SYX-normalized expression is quantified in the graph. Results are mean±s.e.m. (*n*=200). ***P<0.001 (ANOVA).

SYX (also known as Syx1a) and Cysteine-string protein (CSP) are two presynaptic vesicular proteins. It has been recently reported that their downregulation is an early event of dTDP-43 dysfunction *in vivo*; in fact, the expression of these proteins was found to be significantly altered in the heads of our dTDP-43-null fly model TBPH^Δ23^ and in neuromuscular junction (NMJ) presynaptic boutons in muscle 6 and 7 of third-instar larvae (Romano et al., 2014). Starting from these observations, we verified the endogenous dTDP-43 function in *Drosophila* expressing the AggIn transgene. In particular, we compared, by western blotting, the expression of SYX and CSP proteins in the heads of elav-Gal4>UAS_5A versus elav-Gal4>UAS_Egfp control flies. These proteins appeared to be significantly downregulated in our transgenic model (Fig. 7A). Interestingly, the drop in expression was found at all three time points assayed (day 3, 7 and 11), in agreement with the observation that this fly line already has a severe phenotype by 3 days after eclosion.

### Confocal analysis of SYX protein expression in larval NMJs presynaptic boutons

In order to support the hypothesis that endogenous dTDP-43 loss of function was also responsible for the phenotype observed during the larval stage in elav-Gal4>UAS_5A line, we analyzed SYX protein expression in the NMJ presynaptic boutons in muscle 6 and 7 of these transgenic larvae by immunohistochemistry. As expected, SYX levels were strongly reduced in the synaptic terminals of elav-Gal4>UAS_5A third-instar larvae compared to elav-Gal4>UAS_Egfp controls (Fig. 7B, both anti-SYX and merge). By contrast, the anti-HRP staining of the neurons did not highlight any alteration (Fig. 7B, anti-HRP antibody), confirming the specificity of the SYX downregulation in elav-Gal4>UAS_5A flies. Furthermore, the shape of motoneuron terminals at the NMJ did not appear altered in the transgenic larvae versus control.

To provide evidence that the N-terminal portion of TDP-43 is crucial to enhance the trapping of endogenous dTDP-43 in an insoluble non-functional form, we also included elav-Gal4>UAS_Egfp-12×Q/N larvae in the analysis, which do not exhibit any locomotion impairment during the larval stage (Cragnaz et al., 2015). This allowed us to perform a side-by-side phenotypic comparison of EGFP-12×Q/N and AggIn at the NMJ: as expected, and in contrast to what is observed in elav-Gal4>UAS_5A larvae, the EGFP-12×Q/N line did not show any reduction of the SYX levels in the synaptic terminals (Fig. 7B), thus supporting the role ascribed to the N-terminal region of TDP-43.

Consistently, the observation that dTDP-43 functional loss is detected in elav-Gal4>UAS_5A but not in elav-Gal4>UAS_Egfp-12×Q/N larvae correlates with the ability of aggregates to sequester endogenous dTDP-43. Indeed, we recently demonstrated that the climbing impairment in elav-Gal4>UAS_Egfp-12×Q/N flies overlaps with an age-related physiological drop of dTDP-43 (Cragnaz et al., 2015). In order to demonstrate that a more efficient dTDP-43-trapping ability of the AggIn construct mediates the earlier onset of degenerative effects observed in this novel animal model, we analyzed the correlation between phenotype onset and endogenous dTDP-43 levels in elav-Gal4>UAS_5A flies. In this transgenic line, western blot experiments confirmed the same trend of endogenous dTDP-43 physiological drop observed during aging in wild-type flies (Fig. S3). Interestingly, whereas the phenotype onset in elav-Gal4>UAS_Egfp-12×Q/N flies matches with a strong decrease of endogenous dTDP-43 at day 10 (Cragnaz et al., 2015), the elav-Gal4>UAS_5A flies already show an evident climbing impairment at day 3 after eclosion (Fig. 5), a time-point by which dTDP-43 expression has already started to drop, as the decrease in its levels appears to occur in at least two main steps (see day 3 and day 10 in Fig. S3; compare with dTDP-43 expression at day 1), although it is significantly higher than at day 10.

## DISCUSSION

In the past decade several lines of evidence have shown that TDP-43 plays a key role in the pathogenesis of several neurodegenerative disorders, including ALS and FTLD (Neumann et al., 2006; Van Langenhove et al., 2012; Ling et al., 2013). Notwithstanding the large number of studies devoted to characterization of the molecular mechanisms linking TDP-43 aggregation to neurodegeneration, it is still unclear what the role of TDP-43 and TDP-43-positive aggregates is in disease onset and progression.

However, previous studies have identified the structural determinants of the TDP-43 protein that mediate its self-aggregation and trapping into a non-functional insoluble form: they demonstrated that the C-terminal Q/N prion-like domain is important in the protein aggregation process and that the N-terminal region 1-75 is essential to enhance the trapping of endogenous TDP-43 in the aggregates in a non-functional insoluble form (Budini et al., 2012b, 2015). The sequestered TDP-43 loses its functional capacity, but the aggregates do not show intrinsic significant toxicity in the HEK293 cells (Budini et al., 2012b) or in the *Drosophila* eye, a tissue that does not need TDP-43 for its development (Cragnaz et al., 2014).

Furthermore, whereas the role of the TDP-43 C-terminal part in the aggregation process is well established (D'Ambrogio et al., 2009; Igaz et al., 2009; Yang et al., 2010; Jiang et al., 2013; Wang et al., 2013), the implication of the N-terminal region in a growing number of physiological and pathological functions of the protein has been highlighted only by more recent studies showing that the TDP-43 N-terminus, in particular the first ten residues, appear to play a role not only in RNA recognition (Buratti and Baralle, 2001) and cellular localization (Winton et al., 2008), but also in regulating TDP-43 folding, homotypic interaction, splicing functionality and cytoplasmic sequestration (Zhang et al., 2013). Other studies have also suggested that the TDP-43 N-terminus encodes a new type of ubiquitin-like fold that is involved in binding of nucleic acids and that is normally in equilibrium with an unfolded form: the formation of irreversible inclusions relevant for both physiological and pathological processes might occur when this equilibrium is altered (Qin et al., 2014). Finally, we have recently reported that the elimination of the first 75 residues of TDP-43 N-terminus reduces the efficiency of intracellular aggregates to interact with and sequester endogenous TDP-43 (Budini et al., 2015). In fact, the insertion of the N-terminal sequence upstream of an artificial repetition of the Q/N-rich region of TDP-43 (12×Q/N) results in a chimeric protein that induces aggregation and alters the splicing pattern of POLDIP3, a commonly used reporter of TDP-43 dysfunction. This experiment demonstrated that the TDP-43 N-terminal is crucial for efficient sequestration of the endogenous TDP-43 within the inclusions (Budini et al., 2015).

Taking all the above points into consideration, we have now optimized an aggregation inducer with minimal TDP-43 sequences and tested it both in HEK293 cells and in *Drosophila*. This novel construct Flag-TDP-Δ1-ΔC-RRM2F/L-12×Q/N was named, for simplicity, AggIn. We show here that AggIn is able to trigger aggregation, to deplete nuclei of endogenous TDP-43 and to induce loss of the splicing function of endogenous TDP-43 in HEK293 cells (Figs 1B and 2). Importantly, it is also able to induce evident survival and behavioral impairments when expressed as a transgene in *Drosophila* neurons. We have studied two transgenic fly lines (UAS_2B and UAS_5A) with different levels of the AggIn transgene expression. This characteristic was useful to model differential levels of the TDP-43 aggregation process. In fact, only the elav-Gal4>UAS_5A line (with double the AggIn expression of elav-Gal4>UAS_2B) showed motility impairment at the larval stage, whereas both the elav-Gal4>UAS_5A and elav-Gal4>UAS_2B lines resulted in a reduced lifespan and impaired climbing ability during adulthood. These latter effects were more severe when the aggregation was more efficient. In fact, lifespan was reduced to about a quarter (elav-Gal4>UAS_5A line) and a half (elav-Gal4>UAS_2B line) with respect to the median survival of control elav-Gal4>UAS_Egfp flies. Importantly, these effects were observed at a physiological growth temperature (25°C).

These results suggest that the stronger effects observed in the UAS_5A line are due to the higher levels of AggIn expression and support the hypothesis of a direct correlation between efficiency of endogenous dTDP-43-trapping and onset, as well as severity of the phenotype. Interestingly, the higher transgene expression in the elav-Gal4>UAS_5A line was able to trigger a very early locomotion impairment, quantitatively comparable to that observed in the dTDP-43-null TBPH^Δ23^ larvae (Feiguin et al., 2009). In addition, the genotype–phenotype studies were complemented by the observation that dTDP-43 function is lost. This supports the hypothesis that the effect of aggregation is a depletion of functional endogenous dTDP-43. In fact, the levels of CSP and SYX dropped significantly in the heads of elav-Gal4>UAS_5A flies and the latter was also strongly reduced in the larval NMJ compared to elav-Gal4>UAS_Egfp controls. These results suggest that the expression of the AggIn construct in *Drosophila* causes biochemical outcomes associated with endogenous TDP-43 loss of function. These observations are consistent with the results derived from the stable AggIn transgenic cell line HEK293, demonstrating that the transgene expression causes the formation of aggregates able to trap endogenous TDP-43 in a non-functional form. In conclusion, we generated a novel transgenic *Drosophila* line to model TDP-43 aggregation *in vivo* and demonstrated that aggregation contributes to the onset of neurological impairments through a TDP-43 loss-of-function mechanism.

We cannot exclude a minor contribution of AggIn aggregates to neurotoxicity (as suggested by a modest reduction of vision, as shown in Fig. 4B). However, the observation that protein aggregation caused a decrease of presynaptic markers, whose expression requires presence of functional TDP-43 (Romano et al., 2014), strengthens the idea that loss of function of TDP-43 is involved in the pathogenesis of neurodegenerative disorders.

In comparison with other models based on the overexpression of fully functional TDP-43 variants to trigger neurodegeneration (Walker et al., 2015), our aggregation model is based on the expression of a minimal construct containing specific TDP-43 subdomains, which lacks functional RRMs and thus mimicks a splicing-defective version of TDP-43. This greatly limits the risk of possible intrinsic effects of the transgene activity. These novel transgenic *Drosophila* models could help to gain more insight into the molecular mechanisms underlying neurodegeneration and provide a valuable system to test potential therapeutic agents able to prevent, counteract or slow down disease progression by increasing aggregate clearance or by preventing the capture of endogenous TDP-43.

## MATERIALS AND METHODS

### Expression plasmid and stable cell line generation

The AggIn (Flag-TDP-Δ1-ΔC-RRM2F/L-12×Q/N) plasmid was generated by site-directed mutagenesis using the pcDNA5/FRT/TO-Flag-TDP-12×-Q/N plasmid (Budini et al., 2015) as a template. The final construct included: an N-terminal Flag tag, the amino acid stretches 2-100, 173-190, 191-264 (with phenylalanine in positions 229 and 231 mutated to leucine) of the human TDP-43 protein, and 12 repetitions of the human TDP-43 amino acids stretch 331-369 (referred to as 12×QN). Fig. 1A recapitulates the main features of the construct.

For generation of stable cell lines, HEK293 flip-in cells were grown in DMEM-Glutamax-I (Gibco-BRL, Life Technologies Inc., Frederick, MD) supplemented with 10% fetal bovine serum (Gibco-BRL) and antibiotic and antimycotic stabilized suspension (Sigma-Aldrich, St Louis, MO). Cells were transfected using Effectene Transfection reagent (QIAGEN, Inc., Gaithersburg, MD) following the manufacturer's instructions. Co-transfection of 0.5 μg of plasmid together with 0.5 μg of pOG44 (Thermo Fisher, Scientific, Waltham, MA) vector allowed recombination in the genome of the cells. After co-transfection, cells were grown in DMEM-Glutamax-I supplemented with 10% fetal bovine serum with antibiotic and antimycotic until they reached 80% of confluence. The stable integration of the plasmid was then gradually selected using 100 μg/ml Hygromicin B (Gibco-BRL) and 10 μg/ml of Blasticidin (Gibco-BRL). Once cells were selected, expression of the protein was achieved by adding 1 μg/ml of tetracycline (Sigma-Aldrich) to the culture medium.

### Splicing assay

For the splicing assay, 5×10^5^ Flag-TDP-43-WT, Flag-TDP-43-12×Q/N and AggIn cells were seeded in 6-well plates and induced with tetracycline for 72 h. Uninduced cells were used as a control. After induction, cells were collected and RNA was extracted using Trifast reagent (Euroclone, Milan, Italy) according to the manufacturer's instruction. Reverse transcription was performed using M-MLV Reverse Transcriptase (Gibco-BRL) following the manufacturer's protocol. A PCR with TAQ DNA Polymerase (Roche Diagnostics, Mannheim, Germany) was performed for 35 amplification cycles (95°C for 30 s, 55°C for 30 s, 72°C for 30 s) to amplify POLDIP3, BIM and MADD cDNAs. The primers used to test the splicing pattern of POLDIP3, BIM and MADD endogenous genes were: POLDIP3 forward (5′-GCTTAATGCCAGACCGGGAGTTGGA-3′); POLDIP3 reverse (5′-TCATCTTCATCCAGGTCATATAAATT-3′); BIM forward (5′-TCTGAGTGTGACCGAGAAGG-3′); BIM reverse (5′-TCTTGGGCGATCCATATCTC-3′); MADD forward (5′-GACCTGAATTGGGTGGCGAGTTCCCT-3′); MADD reverse (5′-CATTGGTGTCTTGTACTTGTGGCTC-3′).

### Protein expression and immunoblotting of HEK293 stable cell lines

Protein expression of the AggIn construct was analyzed in 5×10^5^ cells seeded in 6-well plates and induced for 72 h. Uninduced cells were also seeded as control. After induction, cells were collected and lysed with 100 μl of RIPA lysis buffer (50 mM Tris-HCl pH 7.4, 150 mM NaCl, 1% NP-40, 0.1% SDS, 1 mM EDTA pH 8, 1 mM PMSF and 0.5% sodium deoxycholate) supplemented with Complete protease inhibitor cocktail (Roche Diagnostics, Mannheim, Germany). Cell lysates were incubated at +4°C for 30 min, then lysed by sonication and centrifuged at 500 ***g*** at +4°C for 5 min. Total protein amount in cell lysates was then quantified by the Bradford method, and 20 μg was loaded in a 10% SDS-PAGE. An anti-Flag (1:1000, catalog number F1804; Sigma-Aldrich) primary antibody and a HRP-labeled anti-mouse-IgG (1:2000, catalog number P0260; DAKO, Glostrup, Denmark) secondary antibody were used for protein detection. Western blotting using a primary anti-POLDIP3 (1:1000, catalog number 5439; Cell Signaling Technology, Beverly, MA) antibody and a secondary HRP-labeled anti-rabbit-IgG (1:2000, catalog number P0448; DAKO, Glostrup, Denmark) antibody was also performed to detect POLDIP3 isoforms.

### Immunofluorescence microscopy

For indirect immunofluorescence, 5×10^5^ HEK-AggIn cells were seeded on slides and induced with tetracycline for 72 h. Non-induced cells were also seeded as a control. Immunofluorescence was performed as previously described (Ayala et al., 2008). As primary antibodies, an anti-Flag (1:200, catalog number F1804; Sigma-Aldrich) and an anti-TDP-43 (1:1000, catalog number 10782-2-AP; ProteinTech, Chicago, IL) were used. The secondary antibodies were anti-mouse-IgG conjugated to Alexa Fluor 594 and anti-rabbit-IgG conjugated to Alexa Fluor 488, and TO-PRO3 dye was used for nuclei staining, all purchased from Life Technologies. Cells were then analyzed on a Zeiss LSM 510 Meta confocal microscope.

### Fly stocks

AggIn and EGFP constructs were cloned in the pUASTattB vector and subsequently sequenced. The constructs were used to create transgenic flies by standard embryo injections (BestGene Inc., Chino Hills, CA, USA). Transgenes were subsequently balanced on the required chromosome to obtain fly stocks. w^1118^ and elav-Gal4 flies were supplied by the Bloomington *Drosophila* Stock Center at Indiana University. Flies were fed on standard fly food (agar 6 g/l; sugar 41.6 g/l; yeast 62.5 g/l; cornmeal 29 g/l; propionic acid 4.1 ml/l), and were maintained and crossed in a humidified incubator at 25°C with a 12-h-light–12-h-dark cycle.

### Lifespan

Adult flies were collected for 2 days from eclosion and transferred to fresh food tubes in a 1:1 male:female ratio (20 total flies/tube). At the third day, death events were scored and viable flies were transferred to fresh tubes. The same was done every 3 days. Survival proportions were plotted as percentage of living flies against days. More than 120 flies were tested for each genotype.

### Phototaxis assay

The assay was performed as previously described (Cragnaz et al., 2014). Briefly, individual flies from each genotype were introduced into the stem of a Y-maze with one arm exposed to violet light (400 nm) and the second arm completely in the dark. The number of flies that moved into the illuminated chamber within 1 min was determined. At least 50 flies per experiment for each genotype were tested.

### Climbing assay

Age-synchronized cohorts of flies were transferred without anesthesia to a 50-ml glass cylinder, and tapped to the bottom with cotton. After a period of adaptation of 30 s, the climbing ability of flies was quantified as number of animals that reached the top of the cylinder (10 cm) in 15 s. Flies were assayed in batches of 20 (1:1 male:female ratio) and the test was repeated three times for each batch of animals. More than 120 flies were tested for each genotype. The number of flies that reached the top was converted into a percentage value, and the mean±s.e.m. percentage was calculated for at least six experiments.

### Larval movement

Wandering third-instar larvae were selected, gently washed and transferred to a Petri dish (0.7% agarose in distilled water). After a period of adaptation (30 s), the peristaltic waves were counted within 2 min. At least 20 larvae were assayed for each genotype. The median number of peristaltic waves performed in 2 min by each genotype was plotted on a graph (mean±s.e.m.).

### Immunoblotting of fly head samples

*Drosophila* heads were homogenized in lysis buffer [10 mM Tris-HCl, pH 7.4, 150 mM NaCl, 5 mM EDTA, 5 mM EGTA, 10% glycerol, 50 mM NaF, 5 mM DTT, 4 M urea and Complete protease inhibitor cocktail (Roche Diagnostics)]. Proteins were separated by 8% SDS-PAGE, transferred to nitrocellulose membranes (Whatman, Clifton, NJ, USA), blocked overnight in a 5% non-fat dried milk solution and probed with the following primary antibodies: rabbit anti-*Drosophila* TDP-43 (1:1500, made in-house), mouse anti-SYX 8C3s (1:2500, Developmental Studies Hybridoma Bank, DSHB, Iowa City, IA, USA), anti-CSP2c (1:9000, DSHB), mouse anti-tubulin CP06 (1:4000, Calbiochem, San Diego, CA, USA) and mouse anti-FLAG M2 (1:1000, Sigma-Aldrich) antibodies. The membranes were incubated with the secondary antibodies HRP-labeled anti-mouse-IgG (1:1000, Thermo Scientific, Rockford, IL, USA) or HRP-labeled anti-rabbit-IgG (1:1000, Thermo Scientific). Finally, protein detection was performed with Femto Super Signal substrate (Thermo Scientific) for anti- TDP-43 and anti-CSP2c immunoblotting and with ECL western blotting substrate (Thermo Scientific) for anti-syntaxin and anti-tubulin antibodies.

Protein expression was quantified using the NIH ImageJ software (Schneider et al., 2012) and normalized against tubulin. Histograms are representative of three independent experiments.

### Solubility test

The solubility assay was performed as previously described (Cragnaz et al., 2014). Briefly, 24 adult fly heads per genotype were homogenized in RIPA buffer [50 mM Tris-HCl pH 8, 150 mM NaCl, 2 mM EDTA, 1% NonidetP40 (v/v), 0.1% SDS, 1% Na-deoxycholate and a cocktail of Complete protease inhibitor cocktail (Roche Diagnostics)]. Following incubation on a rotating wheel for 1 h at 4°C, samples were centrifuged at 1000 ***g*** for 10 min at 4°C. An aliquot was taken at this point as the input, and after a further centrifugation step at 100,000 ***g*** for 30 min at 4°C, the supernatant was collected as the soluble fraction. The resulting pellet was re-suspended in urea buffer (9 M urea, 50 mM Tris-HCl pH 8, 1% CHAPS and Complete protease inhibitor cocktail) and collected as the insoluble fraction. Proteins were separated by 10% SDS-PAGE. The different samples were loaded in a 1:1:1 ratio for the input, soluble and insoluble fractions. Proteins were immunoblotted as already described for fly head samples and probed with the following reagents. Primary antibodies: mouse anti-FLAG M2 (1:1000, Sigma-Aldrich), rabbit anti-GFP (1:2000, sc-8334, Santa Cruz Biotechnology Inc., Dallas, TX) and mouse anti-tubulin CP06 (1:4000, Calbiochem, San Diego, CA) antibodies. Secondary antibodies: HRP-labeled anti-mouse-IgG (1:1000, Thermo Scientific) or HRP-labeled anti-rabbit-IgG (1:1000, Thermo Scientific).

### NMJ immunohistochemistry and images quantification

Third-instar larvae were selected, briefly washed in water and dissected in saline solution (0.1 mM CaCl_2_, MgCl_2_ 4 mM, KCl 2 mM, NaCl 128 mM, sucrose 35.5 mM and Hepes 5 mM pH 7.2), fixed for 20 min in 4% paraformaldehyde, washed in PBS with 0.1% Tween 20 and blocked with 5% normal goat serum (Vector Laboratories, Burlingame, CA) in PBS with 0.1% Tween. Primary antibodies [anti-HRP (1:150, Jackson ImmunoResearch Lab, West Grove, PA, USA) and anti-SYX 8C3s (1:15, DSHB) antibodies] were incubated overnight at 4°C and then the secondary antibodies, Alexa-Fluor-488-conjugated anti-rabbit-IgG and Alexa-Fluor-555-conjugated anti-mouse-IgG (1:500, Thermo Fisher Scientific), were incubated for 2 h at room temperature. SlowFade Gold (Gibco-BRL) was used for the mounting. Images were acquired on a Zeiss LSM 510 Meta Confocal Microscope with a 63× oil lens and 40× lens, then analyzed using NIH ImageJ software (Schneider et al., 2012).

The larvae analyzed for these experiments were processed simultaneously and the same microscope settings were employed to acquire all images. The presynaptic terminals of second abdominal segment on muscle 6 and 7 were analyzed. The samples were double labeled with anti-HRP and anti-SYX antibodies: the mean intensity of both was quantified and a ratio calculated (adapted from Thomas et al., 1997). The statistical analyses were performed using Prism6 (GraphPad, San Diego, CA, USA).
